# Supplementary material for: Are carbon emissions trading and green financial instruments synergistic? -Comprehensive quantitative research based on content analysis
Source: PLoS One. 2024 Mar 7;19(3):e0298601. doi: 10.1371/journal.pone.0298601 (PMC10919601; doi:10.1371/journal.pone.0298601)
Supplement: S1 Data — (ZIP) [file pone.0298601.s001.zip › Data/地级市Z_F工作报告.zip!/╔╜╬≈/╘╦│╟╩╨/└┤╫╘╣1⁄2╓┌║┼í╛┬φ┐╦╩2╛▌═°í┐_2017.docx]

各位代表：

现在，我代表市人民政府向大会报告工作，请予审议，并请市政协委员和其他列席人员提出意见。

一、过去一年工作回顾

2016年是换届之年，全市上下深入学习贯彻党的十八大、十八届三中、四中、五中、六中全会精神和习近平总书记系列重要讲话精神，认真落实中央新发展理念和省委“一个指引、两手硬”重大思路和要求，坚定信心、攻坚克难，凝心聚力、开拓奋进，全市经济社会发展取得了新进展、新成效，实现了“十三五”良好开局。

换届以来，在市委的坚强领导下，在市人大、市政协的监督

支持下，市政府按照中央、省各项决策部署，紧紧围绕建设“三市一中心”目标，以推进供给侧结构性改革为主线，深入实施“三动三新”战略，全力以赴稳增长、促改革、调结构、惠民生、防风险，积极有效挖潜力、增优势、破难题、补短板、打基础，在经济下行压力持续加大的形势下，全市经济呈现企稳向好态势。2016年，全市生产总值完成1222.3亿元，增长4%；规模以上工业增加值完成226.3亿元，增长2.3%；固定资产投资完成1483.7亿元，增长8.2%；社会消费品零售总额完成704.7亿元，增长6.6%；财政总收入完成106.1亿元，增长0.6%；一般公共预算收入完成59.1亿元，增长5%；外贸进出口总额完成80.1亿元，增长9.2%，其中，出口21.4亿元，增长4.6%；城镇居民人均可支配收入完成25636元，增长6.6%；农村居民人均可支配收入完成9365元，增长7.4%。其中，经济总量排名全省第三，固定资产投资、一般公共预算收入和农村居民人均可支配收入三项指标增速排名全省第一，城镇居民人均可支配收入增速排名全省第二。特别是规模以上工业增加值在三季度扭转了连续20个月负增长的被动局面，重回正增长区间，全年完成省定目标任务。城镇新增就业5.04万人，城镇登记失业率2.84%，居民消费价格涨幅1.3%。约束性指标中，劣Ⅴ类水体比例、农村贫困人口脱贫人数、城镇棚户区住房改造数量等指标均完成年度任务。

主要做了以下工作：

一是以“三个一百”为抓手，大力推进实体经济发展，经济增长的质量和效益进一步提升。扎实开展干部入企服务、项目大起底，有效推进“三个一百”工作，解决资金、土地、审批、人才等方面问题652个。重点在建项目超额完成年度投资任务。全年实施招商引资项目385项，到位资金613.9亿元。大力培育新兴替代产业，全年实现增加值107.5亿元，占规模以上工业的47.5%。国澳崇基电梯项目建成投产，芮城光伏领跑技术基地项目开工建设。运城铝工业基地建设扎实推进，与山西铝厂签订了战略合作协议。中铝山西分公司80万吨氧化铝项目全面复产。华圣铝业实现用电直接交易，扭亏为盈。大运重卡集团实现营业收入82.5亿元。建龙钢铁重整海鑫顺利复产。“三去一降一补”效果明显，原煤、焦炭产量分别下降9.9%和6.2%，民生领域投资大幅增长。

二是以现代农业为方向，深入推进农业供给侧结构性改革，“三农”工作进一步加强。全年粮食总产32.15亿公斤，亩产344.5公斤。水果产量58.5亿公斤，蔬菜产量25.8亿公斤。油桃出口澳大利亚，苹果出口加拿大、秘鲁，水果年出口量1.5亿公斤。首届运城国际果品交易博览会成果丰硕，签订97个项目，总投资114亿元。牧原、温氏、大象等畜牧养殖项目顺利推进，良种猪标准化养殖长荣模式得到推广，晋龙蛋鸡规模稳居全省前列。农产品加工销售收入完成252.8亿元。农村土地承包经营权确权登记工作基本完成，县、乡两级全部建立了农村产权流转交易中心。新型农业经营主体健康发展，农民专业合作社和家庭农场总数分别达到12962个和1540家。

三是以培育战略性支柱产业为目标，加快文化旅游产业深度融合，服务业发展进一步提速。举办了首届关公国际旅游节，以“全球华人朝拜圣地，关公故里，大运之城，山西运城欢迎您”为内容的城市形象宣传片，在央视综合频道、新闻频道和中文国际频道播出。着力打造盐池自然景观与关帝庙人文景观相融合的龙头景区，推进关帝庙、李家大院争创5A级景区。永济、芮城被列入第二批国家全域旅游示范区创建名单。认真实施《运城市关圣文化建筑群保护条例》，永乐宫壁画保护工程顺利推进。依托夏县宇达、闻喜本命年两个国家级文化产业示范基地和绛州澄泥砚等省级文化产业示范基地，引领文化产业健康快速发展。2016年，全市旅游人数和旅游总收入均增长30%以上，第三产业实现增加值576.9亿元，同比增长6.4%。

四是以中心城市建设为龙头，完善基础设施和公共服务，城乡一体化进程进一步加快。紧紧围绕建设晋陕豫黄河金三角区域性中心城市的目标，统筹推进中心城市、大县城、小城镇和新农村建设。中心城区围绕城市道路体系、棚户区改造、公共文化和社会服务设施、城市水系等重点领域，实施重点工程40项，累计完成投资11.1亿元。创新城市建设投融资模式，以PPP模式实施的市民服务中心已经开工。编制完成城市水系规划。实施了中心城区集中供热扩网工程，新增热电联产供热面积300万平方米。全市新开工各类保障性住房12394套，基本建成公租房、棚户区改造安置住房11347套，均超额完成省定任务。完成农村危房改造9220户。

五是以改革创新为动力，不断扩大对外开放，发展的活力和动能进一步释放。完成了市县政府部门权责清单编制公布工作，取消、下放行政职权195项。市、区事权关系得到进一步理顺。财政预决算公开范围不断扩大。商事制度改革成效显著，市场主体达到24万户，同比增长25.8%。大力推动农信社改制，盐湖、临猗、永济、河津等10个县（市、区）改制成功并挂牌运营。6家村镇银行开业。积极推进机关事业养老保险制度改革，大力实施全民参保登记计划，城乡居民医保制度整合取得明显突破。城市公立医院综合改革试点工作启动实施，药品加成全部取消。成立了铝及铝加工、镁及镁加工、新能源汽车三个产业技术创新战略联盟。运城科技大市场建设有序推进。全年有效发明专利完成700件，技术合同成交额7.3亿元。F型杂交小麦科研攻关取得重大进展，列入国家重点研发计划。星河众创空间被认定为国家级众创空间。制定出台了《关于化解企业债务风险促进经济社会发展的实施意见》，召开了运城市与驻并金融机构负责人恳谈会，积极破解企业担保链困局，化解金融债务风险。金融业增加值占到地区生产总值的5.9%。企业应急转贷资金规模1.3亿元，累计服务企业150多户次，资金周转额累计达20亿元。“新三板”挂牌企业目前已达到11家，募集资金超过7亿元。运城市产业引导基金总规模达到10亿元。积极推进开发区改革创新发展，明确了改革任务和产业布局。加快发展开放型经济。晋陕豫黄河金三角区域合作第一次省级协调领导小组会议和市级联席会议顺利召开，区域内基础设施实现互联互通，果业合作、文化旅游合作持续增强。运城海关、出入境检验检疫局挂牌后有序运营，海关监管区域建设顺利推进。航空口岸开放已上报国家口岸办审批。

六是以增进民生福祉为目的，持续保障和改善民生，人民群众的获得感和幸福感进一步增强。2016年，全市民生领域支出239.6亿元，占一般公共预算支出的83.3%。168个贫困村、60671名贫困人口实现脱贫。28所新建、改扩建农村幼儿园如期完工，盐湖、新绛、闻喜、河津完成国家义务教育基本均衡县达标验收。中心城区学校建设步伐加快，启动了8所公办学校、2所公办幼儿园建设。其中，5所完全由市财政投资，建成后交盐湖区管理使用。优化整合各类职业教育资源，成立8大职业教育集团，促进了产教深度融合。运城市第一医院项目主体结构完成封顶，市儿童医院综合楼、第三医院住院楼项目开工建设。职业培训各项工作均超额完成任务。城乡低保标准提高。建成130家农村日间照料中心。农村和寄宿制学校公益电影放映广泛开展。群文舞蹈《扫街》荣获全国“群星奖”。完成农村公路建设改造815公里。深入推进环保综合整治，环境质量持续改善，在成功摘牌后，保持了良好的发展态势，二级以上优良天数全年达到252天，比上年增加15天。建立完善灾害性天气预警机制。军民团结、众志成城，成功抵御20年来最大的汾河洪水。对新绛“1·18”粗苯泄露问题处置得当有力，成功避免了一起可能发生的跨省界重大水污染事件，得到了省委、省政府的充分肯定。全市进京非正常上访人次低于年度控制指标，信访事项化解率为93.3%。食品药品安全治理和保障水平持续提升，被列为国家食品安全创建试点城市。安全生产形势持续稳定好转，事故起数和死亡人数实现了“双下降”，全年未发生较大及以上安全生产事故。加强社会治安综合治理，全市未发生重大舆情和重大群体性事件，社会保持了和谐稳定。

过去工作成绩的取得，是省委、省政府和市委坚强领导的结果，是市人大、市政协大力支持、有效监督的结果，是全市上下团结拼搏、共同奋斗的结果。在此，我代表市人民政府向全市人民，向驻运部队、武警官兵和公安干警，向人大代表、政协委员，向所有关心、支持、参与运城改革发展的各民主党派、工商联、无党派人士及各界朋友，表示崇高的敬意和衷心的感谢！

各位代表，在肯定成绩的同时，我们也清醒地认识到，全市经济社会发展还面临不少困难和挑战，有些问题还没有从根本上得到解决，主要表现在：经济下行压力依然较大，2016年全市生产总值、固定资产投资、外贸出口总额未能完成省定考核任务；部分企业因担保链出现问题，债务风险加大，经营困难，金融生态有待进一步优化；招商引资成效不够明显，产业类的大项目、好项目相对较少，支撑带动力不足；文化旅游资源开发利用刚刚起步，资源优势转化为产业优势还需下更大气力；环保形势依然严峻，雾霾问题成为人民群众关注的焦点，水污染防治压力增大，绿色发展、循环发展任重道远；受财力制约，涉及群众切身利益的上学、看病、就业、养老、住房等许多问题亟待解决，贫困村、贫困人口脱贫任务比较艰巨；发展的政务环境还不优，一些部门和干部抓工作力度还不大，一定程度上还存在庸政怠政懒政问题，不作为、慢作为的问题有一定的普遍性；政府系统一些干部的法治思维和法治理念还没有完全树立，运用法治方式解决矛盾问题的能力亟待提升。对这些问题，我们毫不回避，一定全力解决，决不辜负全市人民的期望和重托！

二、2017年工作安排

2017年，是全面贯彻落实省第十一次党代会、市第四次党代会精神的起步之年，是供给侧结构性改革的深化之年。我们要深入学习贯彻习近平总书记系列重要讲话精神和治国理政新理念新思想新战略，认真贯彻中央经济工作会议和省委十一届二次全体会议暨经济工作会议、省“两会”精神，按照市第四次党代会和市委四届二次全体会议暨经济工作会议的总体部署，坚持稳中求进工作总基调，坚持把深化供给侧结构性改革、综改试验区建设与实施晋陕豫黄河金三角区域合作等区域性规划有机结合，坚持以提高发展质量和效益为中心，深入实施“三动三新”战略，加快建设“三市一中心”，全力促进经济稳步向好、民生不断改善和社会和谐稳定，以优异成绩迎接党的十九大胜利召开。

主要预期目标：地区生产总值增长6%，规模以上工业增加值增长3%，社会消费品零售总额增长7%，一般公共预算收入增长3%，城乡居民人均可支配收入分别增长6.5%和7%，居民消费价格涨幅控制在3%左右，城镇新增就业5万人，城镇登记失业率控制在4%以内，固定资产投资增长目标根据新的统计口径研究设置。

约束性指标：资源节约方面，万元地区生产总值能耗下降3.2%，万元地区生产总值二氧化碳排放量下降3.9%，万元地区生产总值用水量下降3.5%。环境质量方面，空气质量优良天数比例、细颗粒物（PM2.5)年平均浓度下降率、地表水监测断面优良水体比例、劣Ⅴ类水体比例不折不扣完成省定任务，化学需氧量、氨氮、二氧化硫、氮氧化物排放总量达到省定要求。民生改善方面，夏县、闻喜两个贫困县如期摘帽，全市208个贫困村、68616名贫困人口如期脱贫，新开工棚户区改造安置房6200套，基本建成棚户区改造安置房9900套。

以上述目标任务为导引，重点抓好以下十方面工作：

（一）紧紧牵牢改革主线

深入推进供给侧结构性改革。大力实施“三去一降一补”。退出煤炭产能30万吨，逐步淘汰容量小、煤耗高、排放不达标、单机20万千瓦以下的火电机组。鼓励有条件的企业走出国门、开展合作。严格控制新建房地产项目审批，棚户区改造全部采取货币化安置，城中村改造货币化安置比例达到80%以上。引导金融机构帮助企业优化债务结构，稳步开展债转股和资产证券化工作，降低企业资产负债率。落实好国家、省惠企减负政策，推进降成本、减负担。建立完善危困企业风险化解机制，鼓励企业加强内部管理，盘活存量资产，实现挖潜增效。统筹城乡一体化发展，补齐教育、医疗、就业、基础设施建设等民生短板。

加快开发区改革创新发展。按照“整合、改制、扩区、调规”的思路，整合5个省级开发区和15个工业园区。运城经济开发区、空港经济开发区合并申报国家级经济技术开发区，盐湖工业园区整合盐湖文化产业园区，申报省级高新技术产业园区，盐湖生态保护与开发区域申报省级生态文化旅游开发区，永济、河津整合工业园区申报设立省级经济技术开发区，绛县经济开发区、风陵渡经济开发区申报扩区。立足各开发区实际，引进和发展能够支撑区域经济快速增长的特色产业。加快建立专业化、市场化、国际化的开发区管理运行机制和领导班子任期制、全员岗位聘任制、绩效工资制，不断增强开发区发展的动力和活力。

深化国企国资和集体企业改革。积极推进党政机关与所办企业脱钩改革。建立完善国有资产监管机制，将城投、水投、热力、机场、南山等公司的国有资产纳入集中统一监管范围。筹备成立交通投资公司、文化旅游投资公司，组建运城投资集团。推进国有企业混合所有制改革试点工作，盘活国有资产。努力解决历史遗留问题，把企业破产清算工作实质性向前推进。全面完成粮油批发市场的改制和新盛外贸公司的整体划转工作。

稳妥推进农村综合改革。深化农村集体产权制度改革，开展集体资产清产核资工作。在全面完成土地确权登记颁证的基础上，有序实施农村土地所有权、承包权、经营权“三权分置”，完善产权交易中心建设，抓好盐湖、新绛农村土地承包经营权抵押贷款试点。稳步推进供销社综合改革，全面推广闻喜惠农服务中心的经验。加快推进国有林场改革，深化集体林权制度改革。以稷山为试点，开展农业水价综合改革。以临猗、稷山为试点，开展水利设施产权制度改革。抓好水权制度改革试点，推行跨区域、跨行业水权转让交易。

扎实推进社保领域改革。落实城乡居民基本医保并轨制度，推进基本医保异地结算和医保支付方式改革。继续推进机关事业养老保险制度改革，实施全民参保登记计划，促进社会保险全覆盖。巩固深化公立医院综合改革，落实政府办医责任，完善医院补偿机制，严格实行药品流通“两票制”，确保药品配送使用安全高效。加快建立分级诊疗制度，大力开展家庭医生签约服务，提升基层医疗卫生机构服务能力，推进县乡医疗卫生一体化，县域内就诊率平均提高到85%左右，基本实现大病不出县。

积极推进“放管服”改革。继续深化行政审批制度改革，及时做好省政府取消下放行政职权的承接落实工作。着力优化审批流程，减少办事环节，推进投资审批网上办理，全面实施随机抽取检查对象、随机选派执法检查人员、抽查情况和查处结果及时向社会公开的“双随机、一公开”监管。加快推进市场监管、卫生计生、文化旅游、商务流通、城乡建设、城市管理6个领域的综合行政执法体制改革，切实提高执法效能。继续削减工商登记前置审批事项，重点做好个体工商户营业执照和税务登记证“两证整合”。推动商标专用权质押贷款工作。

各位代表，改革是生存发展之道，是解决一切发展问题的必然选择。我们一定要认真落实中央、省各项改革政策措施，坚持深化供给侧结构性改革与深化转型综改有机结合，加快推进重点领域和关键环节的改革，坚决打赢改革攻坚战！

（二）深入实施创新驱动、转型升级战略

推动科技创新。围绕发展壮大铝及铝加工、先进装备制造、农副产品加工、现代化工、生物医药、新材料新能源六大产业集群，组织实施一批重点科技计划、应用示范工程和重大产业化项目。深化质量强市，推进品牌建设。深化产学研合作，推进产业技术创新联盟建设。鼓励企业加大技术创新投入，成为技术创新决策、研发投入、科研组织、成果转化的主体。完善鼓励企业自主创新政策，降低企业创新成本，增强企业创新动力。探索运用财政补助机制，激励引导企业建立研发准备金制度。加强高新技术企业培育和认定，建立培育服务机制，落实所得税优惠政策，力争全市高新技术企业数量突破100家。科技大市场一季度投入运营，有效发挥科技成果交易、转化作用。建设以国家级火炬计划特色产业基地和省级科技创新园为引领的创新高地，培育一批产业前景良好的科技型中小企业。加强与中国农大、山西农大、山西农科院、山西中医学院、陕西杨凌高新示范区的对接合作，重点加快F型杂交小麦研发进程，支持双季槐、皂荚、碳能肥等科技创新工程。加强知识产权保护，2017年全市发明专利申请量完成300件，有效发明专利拥有量突破730件。

发展信息技术产业。积极落实网络强国战略，加快全市高速宽带网络和农村及偏远地区4G建设，做好电信普遍服务补偿试点项目，争取2017年宽带家庭普及率达到69%以上，4G移动电话人口普及率达到60%以上，基本建成覆盖城乡、服务便捷、高速畅通、技术先进的宽带网络基础设施。把智慧城市建设落实到具体项目，提升电子政务服务能力。制定大数据产业发展规划，健全大数据产业支撑体系，重点培育和扶持一批龙头骨干企业。以寰烁科技为龙头，大力培育信息制造业、软件和信息服务业，力争形成云计算、教育、医疗、环保、物联网、互联网为核心的产业集聚，不断提升信息产业对我市经济发展的支撑作用。

强化人才支撑。深入实施人才强市战略，建立健全功能完善、运行有序、服务规范的人力资源市场体系，大力引进培养高层次创新型科技人才、新兴产业领军人才、民营企业领军人才、高技能人才。完善户籍、社保、医疗、子女入学等配套政策，建立突出贡献人才生活保障制度，吸引更多优秀人才到我市创业就业。支持非公有制企业、新社会组织引进人才，使体制内外人才享受同等政策。鼓励机关事业单位干部职工到企业驻点帮扶。建立科技工作者智库，实施柔性人才引进机制，鼓励学校、医院、企业与高等院校、科研院所建立多种合作关系。充分发挥我市中高职院校的作用，围绕服务科技创新、产业发展，培育各类专业适用人才。发挥运城在外商会作用，对接全省“晋商晋才回乡创业创新”工程，吸引运城籍在外成功人士、高校毕业生支持家乡、回乡创业、共谋发展。

推进财政金融创新。用好产业引导基金，撬动更多社会资本支持实体经济。转变财政支持方式，增强财政资金使用效益。运用PPP模式，加快推进城市基础设施建设和民生重点领域公共服务设施建设。优化政府融资性担保资源，壮大市级融资担保平台实力。加强社会诚信体系建设，搭建政银企三方沟通对接平台，建立金融债务风险应急处置机制，依法严厉打击恶意逃废金融债务和各类非法金融活动，构建更加良好的金融生态环境。针对重点困难企业，破解担保困局，化解债务风险。加大贷款协调力度，强化银企对接，有效提高银行存贷比。发展多层次资本市场，支持企业上市融资，推动更多企业在“新三板”挂牌。稳妥健康发展互联网金融，引导和培育互联网金融企业，维护金融市场秩序。积极引进更多银行、保险、证券等金融机构落户。

各位代表，创新驱动是运城转型升级的根本出路，人才资源是振兴崛起的关键支撑。我们一定要树立全新的资源观，把创新摆在核心位置，把人才作为第一资源，以科技创新带动全面创新，用全面创新推动经济发展，打造运城未来发展的新引擎！

（三）全面提升对外开放水平

发展外向型经济。编制我市参与“一带一路”建设规划。加强与加工贸易发达地区的沟通联系，主动承接沿海地区加工贸易产业梯度转移。面向国际国内市场，办好运城第二届关公国际旅游节和山西（运城）国际果品交易博览会“一节一会”，把我市的产品推销出去，把国内外游客吸引进来。做好闻喜玻璃器皿、临猗纺织面料出口基地建设。加大国内高端市场推介力度，增加果品直销窗口数量。突出抓好永济、万荣、平陆、芮城、临猗5个国家级出口食品农产品质量安全示范区和新绛国家农产品质量安全示范县建设，完成盐湖、绛县国家级出口食品农产品质量安全示范区创建任务，打造山西重要的农产品出口平台。重点扶持果品出口龙头企业发展，年出口量力争突破1.8亿公斤。积极申报运城跨境电子商务园区试点，推动跨境贸易电子商务发展。落实外贸政策，支持外贸企业发展。建立完善海关、检验检疫、国税和出口企业的联系沟通机制，在出口报关、报检、退税方面提供便利条件，加紧海关监管场所和出入境检验检疫场所申报验收，使更多产品在我市直接出口。运城航空口岸争取前半年临时开放，并同步开展正式开放的准备工作。着手开展综合保税物流中心设计、规划工作。

深化区域合作。制定实施《2017年晋陕豫黄河金三角区域合作行动规划》，加大协调力度，加快推进一批省际交通基础设施、生态治理试点等跨区域重大项目。积极推进运城至三门峡客运专线、闻喜至合阳高速公路临猗黄河大桥等项目前期工作，加紧蒙西至华中铁路、垣曲至渑池高速公路、芮城至灵宝黄河大桥、平陆和风陵渡高速公路出省连接段、108国道黄河金三角段升级改造等项目建设。建立黄河金三角区域产业联盟，做大做强新型铝工业、新型装备制造、新能源新材料和航空产业。加强旅游、果业、物流融合发展，开展文化、体育等交流活动。主动对接、深度融入中原城市群。抢抓政策机遇，积极与相邻市对接，推动一批“铁、公、机、岸、港、网”重点基础设施项目和战略性新兴产业合作项目。

加大招商引资力度。树立系统化、定向化招商思维，变招商引资为选商引资。把招商引资作为各级政府工作的重点，共同负起责任，县（市、区）、开发区、市直产业发展相关部门都要承担相应的招商引资任务，严格考核奖惩，共同推动落实。以开发区为重要平台，围绕重点产业，制定和落实招商引资产业政策和规划。建立招商引资项目库，瞄准重点区域，创新招商方式，实施专业招商、产业链招商、以商招商、以企引企，着力增强招商实效，重点提高签约项目完成率、项目履约率、项目开工率、资金到位率，重点引进世界500强、战略性新兴产业和高新技术产业项目。在招商引资的同时，鼓励支持本土企业扩大投资、新上项目。

各位代表，开放不足是制约运城发展的突出问题。我们要实行更加积极主动的开放战略，坚定不移提高开放型经济水平，以开放激发创新、倒逼改革、推动发展，为运城经济发展拓展新空间、增添新活力！

（四）大力推进工业振兴崛起

培育壮大新兴产业。围绕建设新能源示范城市，加大风电、光伏、生物质等新能源开发利用，推动电能替代，重点支持芮城光伏基地建设。加快重卡及新能源汽车基地建设，大力推进大运新能源与清洁能源汽车及纯电动专用车生产，积极建设大运重卡汽车配套产业园。充分发挥我市电解铝、金属镁的规模和技术优势，加快铝镁合金深加工的发展，努力建设国家级铝镁合金产业基地，重点推进银光镁业500万只轮毂生产线项目。以中车永济电机为龙头，发展轨道交通装备制造产业。推进中药材种植基地建设，支持亚宝、石药银湖、晋新双鹤、朗致、津华、双人等医药企业发展，延伸产业链条，培育壮大医药产业。推动垣曲陶瓷产业发展。更加重视小微企业孵化。支持万荣外加剂、稷山纸包装、闻喜玻璃器皿企业进行资源整合，增强市场竞争力。着力推进通用航空产业发展，打造通用航空全产业链。广泛开展“双创”活动，以企业数量的迅速增加支撑新兴产业的快速发展。

改造提升传统产业。加快运城铝工业“国家新型工业化产业示范基地”建设，以华圣铝业为试点，探索推进局域电网建设，加快推进铝电联营，创新供用电模式，降低用电成本。加快中西部精品钢生产基地建设，推动建龙公司300万吨热轧卷板工程项目投产达效、160万吨窄带钢和工业余热集中供热改造项目建设。支持转型技改项目建设，市财政今年计划列支1亿元专项资金，引导企业开展技术改造。支持大运重卡、建龙钢铁、阳光焦化率先实现年产值超百亿元目标，成为名副其实的大企业。

促进信息化和工业化深度融合。加快复晟铝业智能制造建设，做好亚宝、中铝山西分公司“两化”融合管理体系贯标推广工作，支持大运汽车等大型制造企业、中兴等互联网企业及三大电信运营企业建设“双创”服务平台，培育壮大网络协同制造、个性化定制、服务型制造等网络化生产新模式。

各位代表，运城的工业曾经在全省领先，创造过辉煌的成就。我们一定要提振信心、横下决心，做大工业规模，调优产业结构，增强企业核心竞争力，致力在山西振兴崛起的征程中，走在全省前列！

（五）强力促进农业提质增效

增强粮食保障能力。继续落实“藏粮于地、藏粮于技”战略，加强耕地保护，积极落实耕地质量保护与提升行动，完成耕地质量提升500万亩和高标准农田建设任务。重点推进15个粮食高产万亩示范区创建和5个小麦绿色增产模式攻关项目建设，全面提升粮食综合生产能力，总产量保持在30亿公斤左右。

扩大优质农产品供给。抓住我市果品果业被列为省级战略的机遇，以标准化生产、全流程质量监管、交易平台建设为重点，打造“运城苹果”品牌。实施农业标准化战略，推进无公害农产品、绿色食品、有机农产品和农产品地理标志“三品一标”认证，农业生产标准化率达到30%以上。推进100个精品园和1000个精品专业村“百园千村”建设。发挥果业合作社示范带头作用，加强果农科技培训。推进干果经济林提质增效。积极实施设施蔬菜建设、旧棚改造提升、自动化控制等工程，做强“绛州绿”“夏乐”等果菜品牌，加快发展食用菌产业。

提高规模养殖水平。抓好牧原、温氏、大象等项目建设，开展标准化养殖场示范创建活动，推进畜禽良种化、养殖设施化、生产规范化、防疫制度化、粪便无害化，带动畜牧业提质增效。强化动物疫病防控和畜产品质量安全，逐步构建县乡村（场）三级畜牧业信息化监管网络。

壮大农产品深加工。加快农产品深加工企业的整合、引进，提高核桃、双季槐、皂荚等产品的附加值和市场竞争力。开发功能性食品。扶持壮大万荣汇源、夏县格瑞特、绛县维之王、芮城天之润、垣曲山里红、永济忠民等农产品加工企业，引进一批大企业、大集团，培育一批年销售收入5亿元、10亿元、30亿元的领军企业或企业集团，力争全年农产品加工销售收入完成260亿元。

强化农业基础支撑。加快推进新型农民职业培训、良种良法良田配套集成技术应用、农田果园黄河引水灌溉、主要农作物生产全程机械化、农产品产地市场和网上销售“五个基本覆盖”，全面加强生态保护，全面深化农村改革，全面推进乡村治理，为现代农业发展提供有力支撑。全年完成12000名新型职业农民培训任务。重点培育种养大户、家庭农场、农民专业合作社等新型农业经营主体，发展适度规模经营，新认定家庭农场150家，发展农民专业合作社200家，推动农民专业合作社联合社发展。继续推进北赵引黄二期三期、小浪底引水、部官引水、西范东扩等工程，加强小水网建设，加快末级渠系配套，夯实农业发展基础。提高农业机械化水平，农作物耕种收综合机械化水平达到72.5%。实施农产品市场体系建设工程，规划建设高标准、综合性的市级农产品综合批发市场，逐步打造市县乡三级市场网络体系，畅通销售渠道。

各位代表，运城的农业条件得天独厚，在全省发展格局中举足轻重。我们要发挥优势、率先作为，深入推进农业供给侧结构性改革，加快培育农业农村发展新动能，促进农业增效、农民增收和农村增绿，奋力开创我市农业现代化建设的新局面！

（六）培育壮大文化旅游战略性支柱产业

大力发展文化旅游业。突出关公文化和盐文化主题，连片规划、同步开发，建设盐池自然景观与关帝庙人文景观相融合的龙头景区。支持关帝庙、李家大院创造条件打造5A级景区,整体推进全市景区提档升级。大力引进一批旅游投资商、旅游管理公司、旅游品牌企业，参与我市的文化旅游开发项目。围绕“古中国·新运城”国际旅游目的地和“关公故里、大运之城”城市形象，规划设计精品线路。瞄准国家级主流媒体，加大在黄河金三角区域、通航城市、高铁直通城市、东南沿海地区的宣传推介力度，不断巩固拓展旅游客源地。争取第二届全国青运会在西侯度遗址举行取火仪式，努力扩大运城的影响力。理顺旅游行业管理体制、经营机制和利益关系，完成全市重点景区管理权与经营权分离。以永济、芮城为先导，完善基础设施，加快产业融合，加强市场监管，提升整体服务水平，推动全域旅游发展。大力发展乡村旅游，举办好“花之海·俏运城”美丽乡村游活动。把握好“安、顺、诚、特、需、愉”六字要诀，完善服务体系。推动互联网与旅游融合，促进智慧旅游发展。

加快发展文化产业。培育新型文化市场主体，支持运城制版、宇达青铜、绛州澄泥砚、山西本命年等龙头企业加快发展。以永济、新绛、稷山为重点，培育建设文化产业村。推动绛州鼓乐、高台花鼓、剪纸等非物质文化遗产实现产业化发展，完成10－15个非物质文化遗产传习所命名工作。发展新闻出版、广播影视、文艺事业。加强文化对外宣传交流，推动河东优秀文化走出去，支持集体和个人参加各类全国性的文化竞赛、竞演活动。推动体育产业发展，促进体育消费增长，积极承办国家、省各类体育赛事。

全面加强文物保护。加大文物保护投入力度，市财政列支文物保护经费1500万元。落实人防、技防相结合的要求，各级文物保护单位的保护员全面到位，智能安全综合防护体系尽早建成投入使用，严防出现重点文物失盗、失火、破坏现象。坚定不移推进关圣文化建筑群申遗，切实把永乐宫壁画保护工作做好。推进文物开放单位体制改革试点工作。

提升运城文化软实力。开展“讲文明树新风”“创文明社区，做文明市民”等活动，评选文明单位、文明社区、文明家庭。发挥基层红白理事会作用，推进移风易俗，树立文明乡风村风。实施乡村文化记忆工程，传承发展中华优秀传统文化，将其贯穿国民教育始终，滋养文艺创作，融入生产生活。出版发行《运城市志》，充分发挥资政、存史、教化作用。启动“河东文化系列丛书”编创工作，创作优秀作品，打造艺术精品。加强和改进档案工作，突出档案管理现代化、规范化和信息化建设，提高档案开放利用和服务水平。进一步健全完善市县乡村四级公共文化服务设施，推进基层综合性文化服务中心建设。开展科普宣传，推广科技应用。加大购买公共文化服务力度，继续开展好“戏曲惠民·欢乐百姓”、农村公益电影放映等文化惠民活动。深入开展全民阅读，建设书香运城。建设以社会主义核心价值观、德孝文化、航天精神为内容的主题公园。举办运城解放70周年纪念活动。加强革命遗址保护利用，实施运城烈士陵园和平陆杜马、西牛烈士陵园、夏县堆云洞整修布展工程。深入开展革命传统教育，坚定理想信念，坚定文化自信。

推进商贸物流业发展。制定实施服务业行动计划，突出生产性、生活性和新兴服务业15个重点领域，抓好50个左右的重点项目，年度投资完成100亿元以上。引进和培育商贸流通企业，积极发展城市商业综合体、商业圈、连锁经营等新业态、新模式，加快推进万达广场商业综合体开工建设。推动禹都市场、新绛汾河湾市场改造提升。组织企业申报山西老字号评定工作。开展城市共同配送，提高商贸流通效率。扩大农村消费，加快批发零售住宿餐饮服务特色化、便利化发展。支持永济在外餐饮企业统一标识、统一标准，打造品牌，实现连锁发展。抓好晋陕豫黄河金三角鸿德港储国际物流基地、太铁智慧物流园建设。推动平陆阳煤新科农产品物流工程。大力发展电子商务，深化与龙头电商的合作，开展电子商务进社区、进农村、进景点，争创国家电子商务示范基地。

各位代表，运城历史悠久、人文厚重、文化旅游资源丰富。我们要坚持科学规划、有效保护、合理开发、永续利用，千方百计做好文化旅游资源保护开发这篇大文章，让古老的文化遗存在新时代焕发勃勃生机，惠及全市人民！

（七）加快建设区域性中心城市

推动中心城市率先发展。立足打造精品工程，重点抓好综合场馆、集中供热和垃圾处理、路网系统、地下管廊系统、海绵城市、生态绿化、水务综合治理、学校、医院、棚户区和城中村改造十大类77个项目的建设。加快建设城市路网系统，解决交通拥堵问题，提高通行效率。启动“一环两横两纵”城市快速通道系统工程，今年实施工农街－机场大道、学苑路高架（局部）等23.6公里快速路建设和改造；加快推进跨南同蒲铁路桥梁建设，规划建设过街天桥和地下通道，年内打通12条断头路。高标准、高起点建设东至人民路、西至解放路、南至红旗街、北至河东街区域和南风广场四周区域的城市示范街区，全力打造旧城区的新名片。市民服务中心、老年活动中心、城市规划展览馆、档案馆、图书馆、科技馆、美术馆、大剧院、工人文化宫九大场馆建设全面开工，三年内建成。编制完成《运城市海绵城市专项规划》，做好申报工作，力争列入国家海绵城市建设试点城市。启动“两横三纵五湖连通”水系建设，今年重点实施以樊村水库、安邑水库、八一水库为主体的生态廊道工程和姚暹渠城区段综合整治工程。加快推进垣曲板涧河一级饮用水引入中心城区的建设项目。编制完成《运城盐湖生态文化旅游开发区规划》，推进南山生态修复和绿化、盐池湿地公园、滨湖路、圣惠古镇及盐韵休闲区、关公文化产业园等项目建设。加快推进盐临夏一体化发展进程。加快解决棚户区（城中村）改造存在的遗留问题。

加快城乡一体化进程。加强规划，全面启动县城集中供热供气、城乡安全饮水、县域垃圾集中处理、县城污水处理、农村光纤宽带通信、公共文化场馆、养老服务机构七大类公共服务设施建设，尽快解决城镇基础设施建设滞后、公共服务供给不足等问题。已实现集中供热的县城要扩大供热面积，新绛、芮城、夏县、闻喜、稷山5个未实现集中供热的县城，今年要全面开工。加快永济市、河津市市域副中心城市建设。抓好国家级重点小城镇建设，打造一批经济繁荣、功能齐全、环境优美、辐射力强的中心镇。加快乡（镇、街道办）机关小食堂、小厕所、小澡堂、小图书室、小文体室“五小”设施建设，市财政将对每个乡（镇、街道办）给予10万元补贴，各县财政也要适当给予补贴。深入实施农村人居环境改善工程，加大改气、改水、改厕力度，推动农村生活垃圾治理和生活污水综合利用。启动14个省级、13个市级和一批县级美丽宜居示范村创建工作。加强传统村落保护，重点抓好9个中国传统村落的保护及开发利用。加强农村公路建设，实施总里程400公里的农村公路改造工程。推进农村电网改造升级，完成39个小城镇（中心村）和94个自然村动力电改造任务。发展壮大农村集体经济，支持集体经济破零工程，市财政每年列支1000万元扶持农村集体经济发展。以创建全国文明城市为总抓手，市县两级协同推进国家卫生城市、国家生态园林城市、国家食品安全城市和中国优秀旅游城市创建工作，加强城市管理，提高市民文明素质，提升城市的品位和形象。

各位代表，运城是山西的南大门，区位优越、交通便利，产业多元、政策富集，理应在山西城镇化建设和晋陕豫黄河金三角区域发展中凸显优势。我们要强化产业支撑，完善承载功能，提升文化品位，打造宜居、宜业、宜游的现代文明城市，让全市人民生活得更加美好!

（八）扎实推进生态文明建设

重点抓好大气、水、土壤污染防治。巩固大气污染防治成果。做好“控煤、治污、管车、降尘”工作。加强燃煤锅炉整治，推进散煤清洁化能源替代。加强对钢铁、水泥、焦化等重点行业大气污染防治设施的改造与监管。提升全市油品供应质量，加强对超标排放车辆的管控。强化工地、道路、堆场及大面积裸露地面的扬尘污染控制。加强重污染天气应对，做好监测预警，强化应急措施。全面开展水污染综合防治攻坚行动。严格落实《水污染防治行动计划》，建立以排污许可证为核心的固定源环境管理制度体系，加强对化工、印染、医药等重点涉水行业污染防治设施的改造与监管。“5+15”工业园区全部完成污水集中处理并安装自动在线监控，坚决杜绝工业废水直排。提高中心城区、县城建成区、重点城镇污水收集处置率，提高中水、再生水回用率。开展农村污水处理试点。加强饮用水水源地保护，确保城市集中式饮用水水源地水质达标率达到100%。加快推进夏县白沙河水源地治理。加强地下水生态保护。全面推行“河长制”。严格落实断面生态补偿考核机制，对汾河、涑水河等重点流域不达标断面进行综合整治。启动土壤污染防治工作。开展土壤污染详查，制定《运城市土壤污染治理与修复规划》，着力推进化肥农药使用零增长。完成全市生态红线划定工作，研究探索生态保护红线的管控制度，加强生态保护重要区域日常监控。引深“铁腕治污”行动，依法严惩各类环境违法违规行为。开展领导干部自然资源资产离任审计试点工作。

持续开展造林绿化。以南山生态绿化修复为重点，统筹规划，大力开展植树造林，建设绿色生态屏障，做到经济、生态、观赏为一体，增绿、增效、增收相统一。对盐池禁墙保护区域进行生态标识，建成百里绿廊。完成年度各项造林任务和5个贫困县2.5万亩生态治理任务，全市森林覆盖率提高1个百分点。

大力推进节能降耗。强化节能目标考核，全面推进工业、建筑、交通、公共机构和商贸流通等重点领域的节能降耗工作，推广使用节能产品，确保节能降耗约束性指标顺利完成。在重点领域、重点行业和重点企业，推行先进适用技术，用技术带动企业节能。推广装配式建筑，推动建筑节能和绿色建筑发展，扶持资源综合利用项目。完成市级碳排放交易平台建设。推进建筑垃圾、生活垃圾、餐厨垃圾就地转化、综合利用。

各位代表，建设山清水秀、天蓝地绿的美好家园是我们的共同追求。我们决不以牺牲环境、浪费资源为代价换取一时的经济增长，要像保护眼睛一样保护生态环境，更加自觉推动绿色发展、循环发展、低碳发展，为子孙后代留下可持续发展的“绿色银行”！

（九）全力维护社会和谐稳定

抓好安全生产。压实政府领导责任、部门监管责任和企业主体责任。严格执行安全生产风险隐患排查治理“零报告”制度，按照谁主管谁负责的原则，做到管行业必须管安全、管业务必须管安全、管生产经营必须管安全、管安全生产必须管职业健康。所有企业都要做到安全责任、管理、投入、培训和应急救援“五到位”。推进应急平台系统建设，完善应急预案，做好应急演练，确保应急预案的实效性。整合资源力量，强化统一调度，加强物资储备，建立完善应急救援综合服务体系。突出科技信息支撑，提高安全防控能力。以遏制防范重特大事故为目标，健全隐患排查治理长效机制，发现安全隐患没有及时有效处理的，视同发生安全生产事故进行责任追究。建立安全生产防控体系，加强重大危险源检测监控管理，不断提高危险化学品、矿山企业技术监控水平。建立全市危险化学品监管信息共享平台，将危险化学品生产、储存、使用、经营、运输和废弃处置纳入大数据库。持续开展安全生产大检查，对煤矿、非煤矿山、危险化学品、道路交通、建筑施工、人员密集场所等重点领域开展专项治理，整治非法超限超载运输，做好电梯、压力容器、游乐设施等特种设施安全监管，及时发现和解决问题。充实市县两级安全生产监管执法人员，强化乡镇（街道）执法力量，完善开发区、旅游景区的安全生产监管体制。严格责任倒查制度，实行安全生产目标考核“一票否决”。加强食品药品安全监管，保障人民群众舌尖上的安全。严厉打击矿山、成品油等领域违法行为，规范市场秩序，消除安全隐患。高度重视防震减灾、气象预警和防火、防汛、防疫等工作。完成西塬湖水库大坝加固工程，积极推动汾河下游防洪治理工程。

维护社会稳定。深入开展“平安运城”建设，认真做好征地拆迁、劳动关系、经济金融、交通事故、医患纠纷、环境保护等领域的矛盾纠纷排查化解，妥善处置各类群体性事件，把矛盾化解在基层。加强民族宗教、外事侨务和港澳、对台工作。做好清理拖欠农民工工资的工作，依法严惩恶意欠薪行为，维护劳动者的合法权益。严格落实反恐怖工作责任制，全面深化反恐怖斗争各项措施。始终保持严打高压态势，持续深入推进打黑除恶、铲除黄赌毒、治爆缉枪“三大战役”专项行动。加强网络安全管理，及时发现和打击网络违法犯罪活动。

加强国防后备力量建设。坚持军民融合发展，进一步推进“双拥”工作，规划建设民兵训练基地、武警机动大队新营区。落实现役军人优待、复转军人安置、随军家属就业等政策规定，有效发挥驻运部队在反恐维稳、抢险救灾、应急处突和经济社会发展中的保障作用。做好人民防空工作，全面提升“战时防空、平时服务、应急支援”能力。

各位代表，安全生产连着千家万户，人民生命财产安全高于一切。我们必须认真落实习近平总书记关于总体国家安全观的要求，牢固树立红线意识，时刻绷紧安全生产这根弦，以铁的担当尽责、铁的手腕治患、铁的心肠问责、铁的办法治本，坚决防止重大安全生产事故、重大群体性事件、重大舆情事件和重大金融风险的发生，全力维护来之不易的安全稳定大局！

（十）切实增进民生福祉

全力做好脱贫攻坚。坚持精准扶贫战略，严格落实脱贫攻坚主体责任，落实单位包村、领导包带、工作队到村、干部到户、第一书记到岗和农技人员支撑发展产业“两包三到一支撑”联动帮扶机制。以产业扶贫为支撑，以农民专业合作社为载体，大力实施“八大工程二十个专项行动和七十个具体项目”。提高城乡低保标准，推进脱贫线、低保线“两线合一”。对因年龄、伤残、疾病等失去劳动能力的家庭，实行财政兜底，确保如期脱贫。

保障和改善民生。积极扩大就业，实施职业培训全覆盖计划，统筹抓好农村转移劳动力、城镇失业人员、退役军人、高校毕业生等群体的就业工作。扎实开展就业援助专项行动，托底帮扶就业困难人员，确保零就业家庭动态清零。今年转移农村劳动力6万人。全面落实带薪年休假制度。做好妇女儿童和老龄工作，维护弱势群体切身利益。加强属地管理，确保农村老年人日间照料中心正常运营。发展慈善和红十字会事业，丰富完善社会保障体系。加快社会福利院儿童部前期筹建工作，尽早开工建设。坚持教育优先发展战略，全面推进教育公平，提升各类教育质量，办好每一所学校，继续保持全省领先地位。大力发展普惠性学前教育。推进义务教育均衡发展，区域内义务教育阶段学校校长、教师交流比例分别达到10%，夏县、稷山县完成国家义务教育基本均衡县达标验收。推动高中学校内涵发展，康杰中学、运城中学对口支援夏县、绛县的高中教育。全市新建中小学幼儿园31所，改扩建14所。注重产学对接，大力发展现代职业技术教育，提升职业教育对产业发展的贡献力。支持运城学院建设特色鲜明的应用型地方本科院校。积极推动运城职业技术学院升本和运城师范高等专科学校、运城幼儿师范高等专科学校联合开展升本工作。树立大卫生、大健康的观念，加强卫生与健康事业发展，努力全方位、全周期保障人民健康。抓住《中医药法》实施的机遇，推动中医药事业快速发展。加快推进在建的医疗卫生项目建设，市第一医院建成投入使用。落实社会办医与公立医院同等待遇，支持有条件的非公立医疗机构做大做强。提高基本公共卫生服务财政补助标准。加快医疗机构人才引进和地方人才培养。

今年，省政府提出实施六件民生实事，我市在全面承接、落实到位的同时，进一步扩大规模、提高标准。一是实施5590名残疾预防重点干预和残疾儿童抢救性康复项目；二是对全市所有建档立卡的农村贫困妇女免费进行“两癌”检查；三是为全市城乡怀孕妇女提供免费产前筛查和诊断服务；四是新建282个（省定为70个）农村老年人日间照料中心；五是实施1540名建档立卡农村贫困劳动力免费职业培训；六是免费送戏下乡2000场。在此基础上，我市将再办好五件民生实事：一是实施中心城区及县城建成区集中供热全覆盖工程，今年全面启动，2018年取暖季来临前建成使用，今年继续对中心城区城中村居民清洁能源替代实施财政补贴；二是用两年时间完成城镇所有小街小巷道路平整、路灯照明、视频监控三项工程；三是用两年时间完成农村乡镇垃圾中转站建设任务，今年建成65座；四是中心城区公共自行车系统今年建成运营，建设180个网点，投放5000辆自行车，基本覆盖中心城区范围；五是实现免费法律咨询全覆盖，法律援助工作机构覆盖所有农村、社区及困难企业，做到应援尽援。

各位代表，民之所望，施政所向。让人民过上好日子是政府一切工作的出发点和落脚点。我们要顺应人民群众的关切和期盼，办好民生实事，增进人民福祉，让人民群众拥有更多的获得感和幸福感！

三、加强政府自身建设

以忠诚、法治、担当、创新、高效、廉洁为目标，不断改进工作作风，全力推动各项工作落实，努力建设人民满意的服务型政府。

对党忠诚，坚定正确的政治方向。全面贯彻党的十八大和十八届三中、四中、五中、六中全会精神，深入学习贯彻习近平总书记系列重要讲话精神，认真贯彻省第十一次党代会和市第四次党代会精神，进一步强化政治意识、大局意识、核心意识、看齐意识，不断增强贯彻中央“四个全面”战略布局、省委“一个指引、两手硬”重大思路和要求的自觉性。坚决服从市委领导，积极落实市委各项决策部署，确保政府各项工作始终在党的领导下，沿着正确的方向推进。

依法行政，把政府工作全面纳入法治化轨道。严格遵守宪法和法律，坚持运用法治思维、法治方式开展工作。自觉接受市人大及其常委会监督，坚决执行人大各项决议，积极支持人民政协履行职能，认真办理人大代表建议和政协委员提案。坚持科学决策、民主决策、依法决策，依法制定政府规章和规范性文件，加强决策咨询工作和政府智库建设，实行重大行政决策合法性审查、终身责任追究和责任倒查制度。严格执行统计法规，坚决杜绝统计数据造假，坚决反对虚假招商和虚假投资。加强政府法制机构队伍建设，设立政府法律顾问和公职律师。全面推进政务公开，切实加强行政复议和应诉工作，有效规范行政执法行为。扎实开展“七五”普法，推动落实“谁执法谁普法”责任，加强法律援助和司法救助。坚持把政府活动全面纳入法治轨道、制度框架，确保政府的每一项工作都能经得起法治实践和历史的检验。

主动担当，切实提高政府的公信力。党和人民赋予我们权力，我们就要担当负责、主动作为，在其位谋其事，任其职尽其责，扎实干好每一天，认真做好每件事。坚持讲实话、出实招、办实事、求实效，言出必践，行必有果。新官不仅要创新业，也要理旧事，做不到的不说，承诺的就要兑现，确保政府工作的严肃性、连续性。我们就是要面对困难不退缩，面对挑战不回避，面对矛盾不推诿，用担当的行动诠释对党的忠诚，用为民的情怀赢得群众的信任。

开拓创新，用新发展理念谋划发展、破解难题。进一步解放思想，坚持用改革的精神、创新的思维、开放的理念谋划和推动发展。积极适应改革发展的新形势、新任务、新要求，善于学习和掌握新知识、新观念、新方法，对标发达地区，借鉴先进经验，用足用活政策，破解发展难题。善于把中央、省的各项决策部署同运城实际紧密结合，创造性地开展工作，不断开创各项工作新局面。?

务实高效，不断提升政府工作的执行力和落实力。认真执行“13710”工作制度，加大政务督查力度，确保中央、省、市各项决策部署落地生根。继续坚持问题导向、强化领导包联、严格督查考核，着力解决重点企业经营、项目建设和招商引资中遇到的实际困难，持续推进“三个一百”工作。围绕今年初步确定的268项省、市重点项目，继续落实一名分管领导、一个主责单位、一套工作班子的管理制度。对上级的部署、下级的请示、群众的诉求，都要及时作出回应。要把案无积卷、事不过夜作为常态坚持下去。创优发展环境，加大问责力度，狠抓工作落实，重点解决中间环节存在的办事拖拉、慵懒散慢、漠视群众等问题，努力消除“中梗阻”现象。

清正廉洁，树立人民政府良好形象。各级政府及其工作部门、所属单位党组都要认真履行全面从严治党主体责任，带头执行党章党规党纪，加强和规范党内政治生活，深入推进政府系统党风廉政建设和反腐败斗争。对于重大项目、重要工作，审计部门要提前介入、全程跟踪、严格监督，实现公共资金、国有资源、国有资产和领导干部履行经济责任情况审计全覆盖。加强内部审计，坚持和完善政府部门和直属机构领导干部离岗交接制度。全面加强财政预算管理，完善财政预算绩效管理体系，既要厉行节约，进一步压缩“三公”经费，又要集中财力办大事，确保有效支出。推动落实中央八项规定精神和国务院“约法三章”常态化、长效化，坚决查处隐形变异“四风”问题。积极构建“亲”“清”新型政商关系，敢于和企业家交朋友，主动帮助企业破解发展难题。加强政府系统党组织建设，教育引导党员干部廉洁从政、廉洁用权、廉洁修身、廉洁齐家，做一身正气、两袖清风的模范，用政风带动社风、民风持续好转，树立良好的政府形象。

各位代表，我们的政府是人民的政府，为人民服务是政府的天职，对人民负责是政府工作的准则。我们一定要牢固树立以人民为中心的发展思想，始终站稳人民立场，时刻把人民放在心中最高位置，竭尽全力做好每一项工作，切实履行好肩负的职责和使命！

各位代表，好雨知时节，瑞雪兆丰年。实现转型综改重任在肩，加快振兴崛起时不我待。让我们更加紧密地团结在以习近平同志为核心的党中央周围，在省委、省政府和市委的坚强领导下，上下同欲、和衷共济，只争朝夕、拼搏奋进，撸起袖子加油干，扑下身子抓落实，走好新的长征路，实现新的运城梦，以优异成绩迎接党的十九大胜利召开！
